# Early stage nonclinical pulmonary disorder in COVID-19 may present asymptomatic and fuel the contagion

**DOI:** 10.1186/s40779-021-00316-5

**Published:** 2021-03-24

**Authors:** Kamoru Ademola Adedokun

**Affiliations:** Oral Pathology Unit, Dental University Hospital, King Saud University Medical City, Riyadh, 11545 Saudi Arabia

**Keywords:** Acute respiratory diseases, COVID-19, Dyspnea, Gas exchange, Oximetry, Pulmonary disorder, SARS-CoV-2, Silent hypoxemia

## Abstract

Evidence shows that pulmonary problems in coronavirus disease 2019 (COVID-19) may set off from vascular injury that progresses to physiological disturbances through a compromised gas exchange, following an infection with the severe acute respiratory syndrome coronavirus 2. In this process, inefficient gas exchange in the alveolar could precipitate silent nonclinical hypoxemia. Unfortunately, patients with “silent hypoxemia” do not necessarily experience any breathing difficulty (dyspnea) at the early stage of COVID-19 while the disease progresses. As a result, several asymptomatic, presymptomatic and patients with mild symptoms may escape quarantine measure and thus continue to spread the virus through contacts. Therefore, early diagnosis of “silent hypoxemia”, which attracts no clinical warnings, could be an important diagnostic measure to prevent acute respiratory distress syndrome from the risk of pulmonary failure among the presymptomatic and as a screening tool in the asymptomatic who are hitherto potential spreaders of the virus.

**Dear editor,**

There is growing evidence that pulmonary problems in coronavirus disease 2019 (COVID-19) may set off the damage in the alveolar capillary [1]. Mechanistically, the pathologic process could shorten the volume of the alveolar oxygen intake due to the lung diffusion impairment precipitating hypoxemia and acid-base imbalance, which ultimately leads to acute respiratory diseases (ARDs), if not followed up (Fig. 1).

**Fig. 1:**
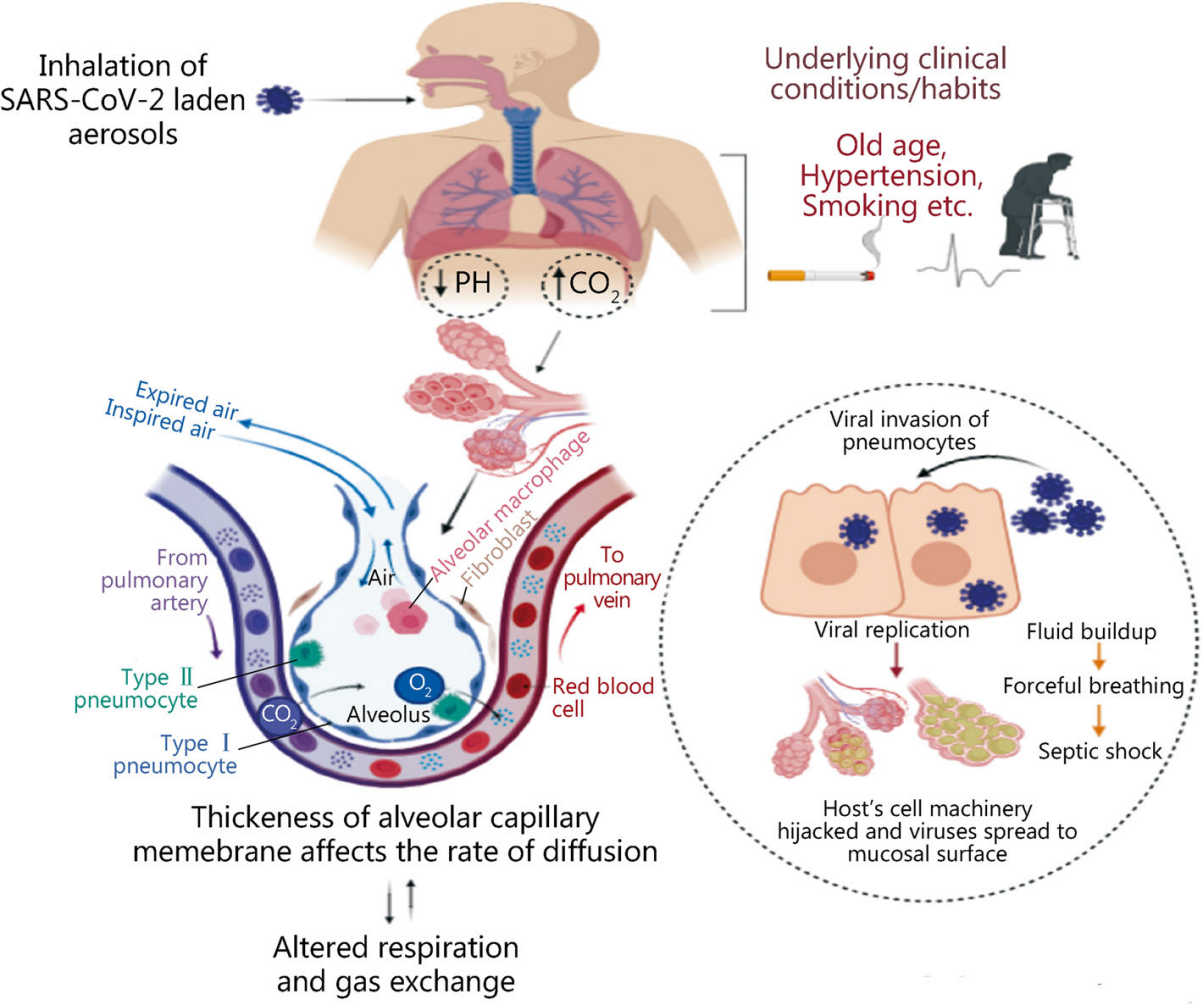
The underlying mechanism in the development of acute respiratory diseases in association with COVID-19

Meanwhile, early severe acute respiratory syndrome coronavirus 2 (SARS-CoV-2) induced pulmonary injury may subsequently aggravate physiological disturbances, even though the patients may remain insensible to any breathing problems. Unfortunately, several patients who advance to pulmonary complications, such as respiratory failure, might have experienced hypoxemia and hypocapnia without any prior signs of respiratory distress. While dyspnea remains one of the criteria for screening and care management, it is worth noting that low prevalence of dyspnea has been repeatedly reported among COVID-19 cases [2, 3]. Currently, an emerging report showed that about two-thirds of COVID-19 patients actually show no sign of dyspnea [2]. The report further revealed that out of 1712 COVID-19 patients under investigation, 1107 patients had no dyspnea-related clinical complaints even at admission, while 757 (68.4%) of these patients revealed clinical signs of pneumonia using low-dose computed tomography (LDCT) scan.

A report showed that many patients with hypoxemia and hypocapnia do actually progress to respiratory failure without any prior signs of respiratory distress [2]. In addition, several reports corroborate that COVID-19 patients may encounter a deadly “silent hypoxia” without clinical warning of the near-danger [4]. From the foregoing, it is becoming evident that several COVID-19 cases do not necessarily show any signs of dyspnea until admission period. However, many nonclinical asymptomatics or preclinical (presymptomatic) hypoxemic patients may continue to fuel the spread of SARS-CoV-2 virus before detection. As a result, there is a need to further studies on potential diagnosis of nonclinical early pulmonary disorder in COVID-19 patients, who might increase the burden of the containment operations. More so, asymptomatic hypoxemia has a poor clinical outcome among COVID-19 cases [2]. Hence, early detection of asymptomatic COVID-19 individuals and particularly the presymptomatic at the early stage of the disease may also prevent the risk of pulmonary failure and thus reduce the menace of global fatality rates associated with COVID-19.

Hypoxemia can be defined as an abnormal deficiency of oxygen concentration in the blood, in terms of the partial pressure of oxygen (mmHg) or the content of oxygen (milliliter oxygen per deciliter of blood). It is a sign of a breathing-related problem or impairment in the blood gas exchange. Diagnostically, asymptomatic hypoxemia could serve as an indicator for screening purposes using various simple, rapid, and noninvasive tests. There are a number of U. S Food and Drug Administration (FDA)-approved diagnostic tools, such as fingertip pulse oximeter (EAD, Concord Health Supply, Skokie, IL) at no cost to the patient [5]. Others are diffusing capacity of the lung for carbon monoxide (DLCO)/lung diffusion test (LDT), spirometry, among others.

The pulse oximeter measures peripheral oxygen saturation (SpO_2_) with a normal range value between 92 and 100%. This device is a painless and portable tool for measurement of arterial oxygen saturation by infrared light refraction to determine oxygen-binding levels to red blood cells, based on differential light absorption properties of oxyhemoglobin and deoxyhemoglobin levels. Some available oximeters, which follow accuracy criteria as provided in the International Standard ISO 80601-2-61 that meet the FDA criteria, could offer diagnostic value for COVID-19.

The previous use of pulse oximeter for screening asymptomatic undetected cardiopulmonary disease showed an acceptable sensitivity, specificity, and predictive value of 75, 99.29, and 99.94%, respectively [6]. Thus, the use of a pulse oximeter could serve as a diagnostic utility in early detection of preclinical silent warning (“silent hypoxemia”) of COVID-19.

Furthermore, Shah et al. [5] suggested that pulse oximetry is an attractive low-risk intervention method that could help detect decompensating COVID-19 individuals. Meanwhile, hypoxemia commonly exists without dyspnea, thus while individuals battling with COVID-19 undergo cardiorespiratory compensation at the outset of hypoxemia, this compensation may not prolong before complications arise [7]. Oximetry was recently put to clinical trial [5], and showed perfect results for identifying patients who needed hospitalization.

While the RT-PCR kit could only perform the detective/screening role and might not offer any benefit as a symptom-monitoring tool, it is believed that pulse oximetry would be beneficial for screening and, in monitoring nonsevere cases against rapid deterioration and possible development of fatal complications. In other words, a portable fingertip pulse oximeter that measures SpO_2_ could also be useful as a symptom checker, where the diagnostic usefulness of RT-PCR is currently limited. It is important to know that addressing the concerns associated with rapid deterioration of hypoxemia is also an urgent need to address the global fatality rates associated with COVID-19.

## Data Availability

Not applicable.

## Abbreviations

ARDs: Acute respiratory diseases
COVID-19: Coronavirus disease 2019
DLCO: Lung for carbon monoxide
FDA: Food and Drug Administration
LDCT: Low-dose computed tomography
LDT: Lung diffusion test
SpO_2_: Peripheral oxygen saturation
SARS-CoV-2: Severe acute respiratory syndrome corona virus type 2
